# Proceedings of the Racine Medical Association

**Published:** 1863-06

**Authors:** John G. Meachem

**Affiliations:** Secretary


### ART. II.—*Proceedings of the Racine Medical Association.*

The Association met at the office of Dr. Hoy, on Tuesday evening the 14th of April—Dr. Page in the Chair.

*Dr. Meachem* read a paper on Albuminuria. He said that Dr. Bright first presented his views to the profession in his "Reports of Medical Cases," published in 1827. His investigations led him boldly to affirm that dropsy depended in almost every instance upon a peculiar degeneration of the structure of the kidneys, which he pleased to call granular, and that these granulations could positively be diagnosticated by the presence of albumen in the urine. It was not necessary for him to enter into a minute description of the symptoms. The bloated body, the waxy skin, the scanty, albuminous and sometimes bloody urine, the pyrexia, the nephritic pains, &c. are generally sufficient to inform us with what we have to contend. There exists, he said, a great tendency for certain inflammatory diseases to supervene, upon an attack of Bright's disease. Pericarditis, pleuritis, bronchitis, arachuitis, pneumonia and rheumatism, he considered the most liable. For treatment he relied mainly upon the hot air bath, sup. tart. potass, iodid potass, digitalis, squills, &c. He had recently treated a case in which he had been enabled to increase the urinary secretion from 16 to 60 ounces in 24 hours, by the use Tr. digitalis and Tr. squills, equal parts.

After the reading of the essay, the President proposed Rheumatism as a subject for the evening's discussion, and called upon Dr. Wadsworth to open.

*Dr. Wadsworth* said that the few remarks he had to offer would be upon the treatment of the disease. He had treated it by a variety of means, and generally with satisfactory results. In the first, and febrile stage of acute rheumatism, he had used the Tr. veratrum viride, with sol. acetate of ammonia and spirits nitre dul. After controlling the pulse and subduing the fever to a considerable extent, by these means, he employed the Tr. colchicum sem. with iodid potass, with marked success. He had also in some cases tried the calomel and opium treatment. After the slightest perceptible effect of the mercury had appeared, he omitted the calomel and substituted quinia with the opium, and in this way had brought severe cases to a favorable termination. He had no experience in the treatment by large doses of nitre, but could readily believe it might be useful, on the same principle that veratrum acted, by controlling the circulation and the fever. He had seen one case recently, recover under Homœopathic treatment, which led him to believe that there might be something in what Watson found to be the *very best remedy*, namely, *six weeks.*—In the chronic form of the disease, he had often found benefit from the following: ℞ Sulphur ʒij, Pulv. G. Guaiac, Pulv. Rad. Cochi, aa grs. xx. Divide into eight equal parts, and give one powder every four hours, until the bowels are thoroughly moved. The above, with an ounce of serpentaria, may be added to a pint of gin, and a table spoonful given three times daily, with good effect. He had known many patients greatly relieved by using this remedy.

*Dr. Hoy* did not think rheumatism a very formidable disease to manage. He had treated it in a great variety of ways, and found results about the same under them all, and therefore he was not wedded, like some practitioners, to any one particular mode of treatment. He believed it should be treated on general principles, and not with the idea that there were any specifics for the disease, for he did not believe there were any. If the fever was high, it should be moderated. If the secretions were suppressed, in any way, they should be freely opened. If the patient suffered much pain, anodynes should be freely administered. Where the rheumatic inflammation was confined to large joints, it was his practice to blister extensively.

*Dr. Meachem* asked him if he had not seen metastasis produced by applying blisters too early?

*Dr. Hoy* did not remember that he had. He did not usually apply them until the active stage of the disease had passed.

*Dr. Meachem* said this was the point he wished to bring out. He did not think it good practice to blister *early* in rheumatism. He had seen translation produced by it in more than one instance.

*Dr. Wadsworth* had also seen the same.

*Dr. Thompson* was quite sure that he had cured many severe cases by liberally bleeding them in the beginning, and then putting them under the full influence of calomel and opium. This, for many years, was the treatment upon which he relied, and he was not willing now to own that there was a much better plan in use.

*Dr. Meachem* thought that our success in managing rheumatism depended very much upon the amount of vigilance we exercise in looking after cardiac complications that occur during its progress. That they are much more frequent than is generally supposed, he had no doubt, and are often times overlooked. He believed them to exist in nearly one-half of the cases. Endo carditis he had found more often than peri carditis, and very frequently both existed together, He did not think these complications resulted so much from metastasis as *by extension*, as he had found very few cases relieved elsewhere when the cardiac disease had manifested itself. Children he thought more liable to these conditions than adults, He had never known an uncomplicated case of rheumatism prove fatal. Its danger always consists in the substitution of some local inflammation. as of the heart, pleura, brain, &c. He had seen cases of simple rheumatism treated in the Bellevue Hospital, entirely on the *placeboic* plan, recover as rapidly as those put under a more active treatment. He had tried the alkaline treatment, and also that by large doses of nit. potass, and of the two he preferred the latter. Anodynes he considered as imperatively demanded in most cases, for the comfort of the patient, and he believed they did as much towards controlling the disease as any other remedy.

*Dr. Nettleton* had great confidence in the alkaline treatment, and he thought the name of Fuller should be placed along side with that of Jenner as a benefactor of our race.

*Dr. Page* said he had been making some trials with the prophylamin. He had selected three excellent cases in which to test the value of the remedy. He had used the article prepared by Nichols of Boston, and also that from a reliable Philadelphia house. No good effect followed the use of either article, in any of his cases. He had given the full dose advised, and had continued it for days together. He was satisfied that it was overestimated as a remedy in rheumatism. He had no confidence in it, from the trials he had made with it, and he believed he had given it every possible chance to act favorably if it possessed any specific powers over the disease. In a recent case that he had treated, where he prescribed the iodid potass rather freely, an erythema of the face was produced, attended with considerable tumefaction. It subsided on omitting the medicine, and re-appeared when it was resumed.

John G. Meachem, M. D., Secretary.

---

Dr. R. J. Levis has retired from the editorship and proprietorship of the Philadelphia *Reporter*, and entered the army as a Surgeon of Volunteers.

